# Supplementary material for: Perturbation Variability Does Not Influence Implicit Sensorimotor Adaptation
Source: PLoS Comput Biol. 2024 Apr 10;20(4):e1011951. doi: 10.1371/journal.pcbi.1011951 (PMC11034674; doi:10.1371/journal.pcbi.1011951)
Supplement: S1 Appendix — (PDF) [file pcbi.1011951.s001.pdf]

Supplementary Information

Supplementary Results

1. The influence of motor variability on estimation of error sensitivity

Consistent with their Memory of Error model (MoE), Albert et al. (2021) [1] report that error sensitivity is higher in the zero variance condition compared to the high variance condition (see Fig 4a in their paper). Superficially, this result appears to be at odd with the Non-Linear Motor-Correction model (NLMC). However, as reported in the main results, our simulations indicate that an estimate of the error sensitivity function cannot be recovered from the block design used in their study. More generally, we believe it is difficult to recover an error sensitivity function in designs in which the effects of learning accumulate. We develop the theoretical basis of this claim in this section.

Error sensitivity for a given trial n, with error size, e is calculated as follows:

$$b(n,e) = \frac{y(n+1)-y(n)}{e(n)} = \frac{x(n+1)+E(\sigma_M)-x(n)-\sigma_M(n)}{e(n)} \text{ [S1]}$$

where $y(n)$ is the movement angle, and $x(n)$ is the state of the internal model on trial n. Consider the situation late in learning where performance is at asymptote with the state largely unchanged from trial to trial ($x(n+1)$ = $x(n)$). When the perturbation is fixed, variation in heading angle is dominated by motor noise (σ_M). Given that the average motor noise by definition is 0, ($E(\sigma_M)$=0), we have

$$b(n,e) = \frac{-\sigma_M(n)}{e(n)} \text{ [S2]}$$

Thus, error sensitivity, $b(e)$, when estimated this way would not reflect the real error sensitivity of the adaptation system. Rather it is function determined by error size and the motor noise for that trial. Given that the average residual error (RE) at asymptote is a constant, we can rewrite S2 as:

$$b(n,e) = \frac{e(n)-R.E.}{e(n)} = 1 - \frac{R.E.}{e(n)} \text{ [S3]}$$

From Eq S3 we see that $b(e)$ does not reflect the error sensitivity of the system; rather, the estimate of $b(e)$ increases as the error size decreases when estimated at asymptote.

We note that the above example comes from the case where learning is saturated. However, the estimate of error sensitivity will be contaminated by this problem at all phases of learning in a block design. It will be especially pronounced when the hand angle approaches the asymptote or when motor noise is large

(Fig D-E in S1 Appendix). The problem is not present in a trial-by-trial design where the mean perturbation is 0° since there will be no accumulated learning. As such, error sensitivity can be reliably estimated (confirmed in our simulations, see Fig Db in S1 Appendix).

2. A hybrid model of implicit adaptation

In response to a preprint we posted that described how the NLCM model provides an alternative account of the effect of perturbation variability, Albert et al. proposed a model in which baseline error sensitivity follows the non-linear motor correction function (a prior) but is modulated by the memory of errors [2]. This hybrid model can predict the large difference between high and zero variance conditions observed early in learning (e.g., around epoch 10), an effect that is not captured by either the NLMC or MoE models (Fig Aa in S1 Appendix).

However, there are some serious limitations with a hybrid model of this form. First, to explain the large difference during early learning, the best-fitted hybrid model predicts a very large hyper learning rate of the error sensitivity (a), which is 0.21 (Fig Ab in S1 Appendix). Given that the model fit results in estimated baseline error sensitivity of 0.04, the model assumes a five-fold increase in error sensitivity after one trial, a rate of changes that seems unreasonably fast (Fig Ac in S1 Appendix). Moreover, the hybrid model predicts a hyper retention rate (b) of 0.19. This would suggest that the change in error sensitivity will only last for a single trial and, thus, result in large instability in error sensitivity across trials (Fig Ac in S1 Appendix). Similarly, the model predicts a marked change of error sensitivity across trials in a trial-by-trial design (Fig 2a in the main text). These predictions fail to conform with the empirical results showing no change in error sensitivity across trials in all conditions.

We note that the large effects on performance that are observed early in learning are sometimes due to the deployment of an explicit strategy [3]. To minimize strategy use, Albert et al. restricted preparation time. However, this manipulation may not completely block the involvement of a re-aiming strategy [4,5], and this issue is likely more relevant in the Zero variance condition than in the High variance condition. Explicit contributions to learning might be more prominent in the former because the evaluation of an aiming strategy would be easier given the fixed perturbation size. Indirect support for this hypothesis comes from the data reported in Experiments 1-3 of Albert et al. In these experiments, there was no limit on preparation time and performance changes can be assumed to reflect contributions from implicit and explicit processes. Under these conditions, the difference between the Zero and High variance conditions

only emerges when performance is near asymptote. We note that this is predicted by the NLMC model since the sampled error at asymptote will span the non-linear zone.

Reference

1. Albert ST, Jang J, Sheahan HR, Teunissen L, Vandevoorde K, Herzfeld DJ, et al. An implicit memory of errors limits human sensorimotor adaptation. Nature Human Behaviour. 2021;5: 920–934.

2. Albert S, Shadmehr R. Perturbation variance suppresses error sensitivity in the implicit learning system. bioRxiv. 2022. p. 2022.09.26.509572. doi:10.1101/2022.09.26.509572

3. Taylor JA, Krakauer JW, Ivry RB. Explicit and Implicit Contributions to Learning in a Sensorimotor Adaptation Task. Journal of Neuroscience. 2014;34: 3023–3032.

4. Haith AM, Huberdeau DM, Krakauer JW. The Influence of Movement Preparation Time on the Expression of Visuomotor Learning and Savings. Journal of Neuroscience. 2015;35: 5109–5117.

5. Huberdeau DM, Krakauer JW, Haith AM. Practice induces a qualitative change in the memory representation for visuomotor learning. J Neurophysiol. 2019;122: 1050–1059.

6. Hutter SA, Taylor JA. Relative sensitivity of explicit reaiming and implicit motor adaptation. J Neurophysiol. 2018;120: 2640–2648.

7. Tsay JS, Avraham G, Kim HE, Parvin DE, Wang Z, Ivry RB. The effect of visual uncertainty on implicit motor adaptation. J Neurophysiol. 2021;125: 12–22.

8. Tsay JS, Ivry RB, Lee A, Avraham G. Moving outside the lab: The viability of conducting sensorimotor learning studies online. Neurons, Behavior, Data analysis, and Theory. 2021. doi:10.51628/001c.26985

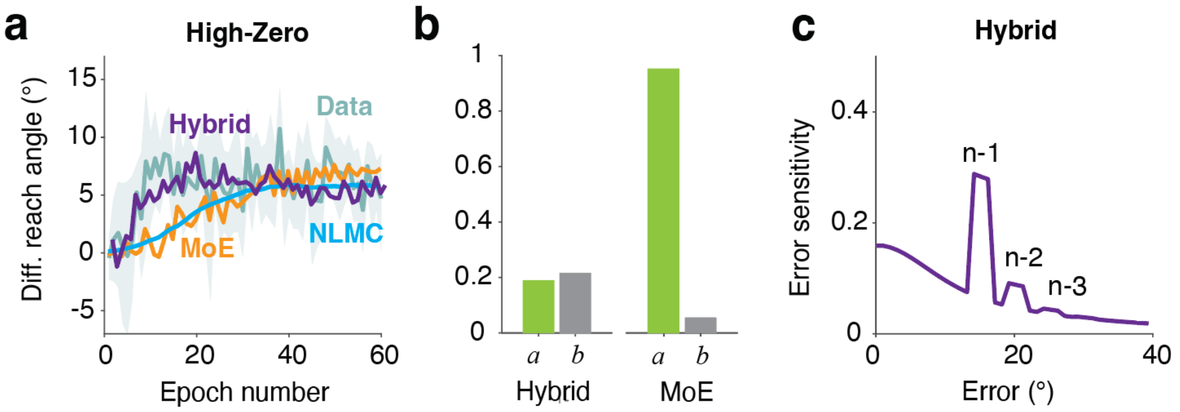

Fig A | Evaluation of a Hybrid model for implicit adaptation. a) The difference between hand angle for the Zero and High variance conditions predicted by the MoE, Hybrid, and NLMC models, along with the data from Exp 6 of Albert et al. (2021) [1] (Shaded area indicates S.E.) In the actual data, the difference peaks early in the learning block (around epoch 10). Only the Hybrid model is able to capture this effect. **b)** However, this is achieved by having a large hyper learning rate (*b*) and small hyper retention rate (*a*, 1-forgetting) for error sensitivity. Not only are these parameter values unrealistic but they are quite different from those estimated for the MoE model. **c)** The Hybrid model predicts a fast change of error sensitivity across trials. In this example, the experienced errors for the first five trials are 30°, 25°, 20°, 15°, and 10°. The error sensitivity calculated for n-1 is much higher than the baseline level because of the high learning rate for error sensitivity (*b*). This effect does not persist when sensitivity Is calculated for trials n-2 and n-3 since the retention rate of error sensitivity is very small.

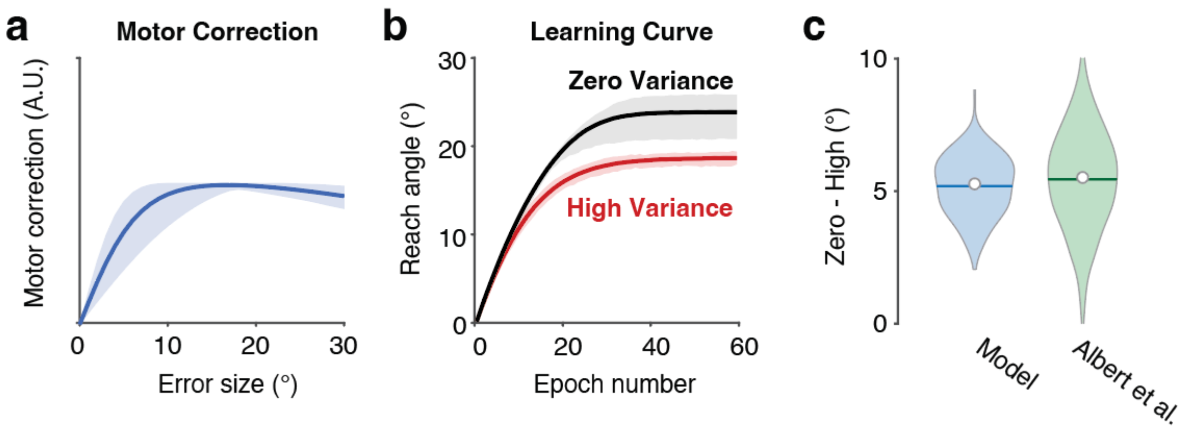

Fig B | The non-linear motor correction function robustly predicts the effect of perturbation variability. To estimate the 95% confidence intervals for the simulation shown in Fig 1, we used a bootstrap procedure. We took 24 samples (with replacement) of the data from our pool of 24 participants, estimated the non-linear motor correction function, and repeated this procedure 200 times. **a)** The normalized best-fitted motor correction function. The peak of the function varied from 6-18°. The solid line shows the mean, and the shaded area shows the 95% confidence interval. **b)** Simulated learning curves for when the experienced error has zero or high variance. Importantly, the 95% confidence intervals for the zero and high variance conditions do not overlap. **c)** The difference between the two conditions at asymptote is very similar for the model simulations and results reported in Albert et al. (2021) [1].

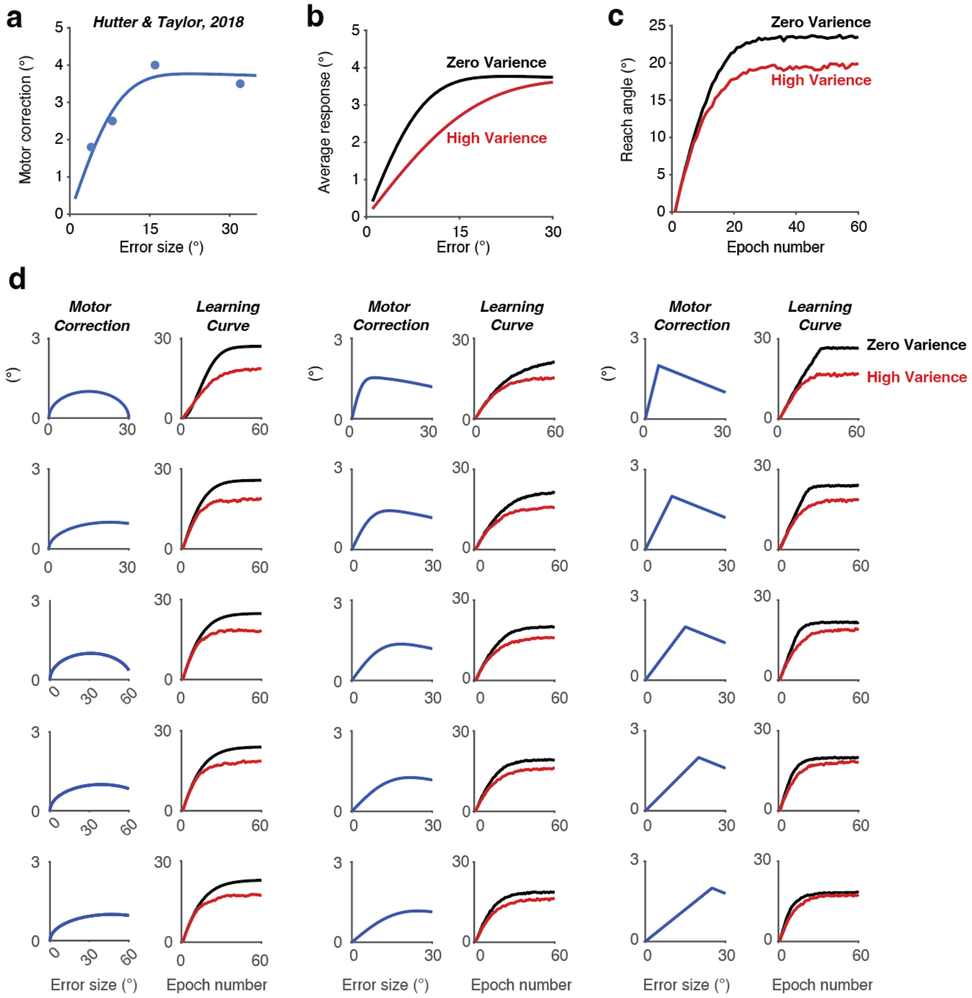

Fig C | The NLMC model captures the effect of error variability when using a motor correction function derived from Hutter and Taylor (2018) [6] **as well as range of hypothetical motor correction functions.** **a)** Size of the motor correction as a function of the error experienced on the previous trial. Dots correspond to sampled values in Exp. 2 of [6]. Solid line indicates the best-fit model. **b**) Average motor correction as a function of mean error size under low (black) and high (red) perturbation variability conditions. **c)** Simulations of the NLMC model showing estimate of implicit adaptation. **d)** Effect of perturbation variability holds across a range of hypothetical motor correction functions. For each subplot, the motor correction function is depicted on the left and simulations of the learning functions for High and Zero variance conditions are shown on the right. While the magnitude of the effect varies, the difference between the high and zero conditions holds regardless of the peak position, with adaptation attenuated in the high variability condition (red) relative to the zero variance condition (black).The one exception is the condition shown in the lower right, a situation in which the function is essentially linear over the range of errors that would be experienced in Albert et al. (2021) [1].

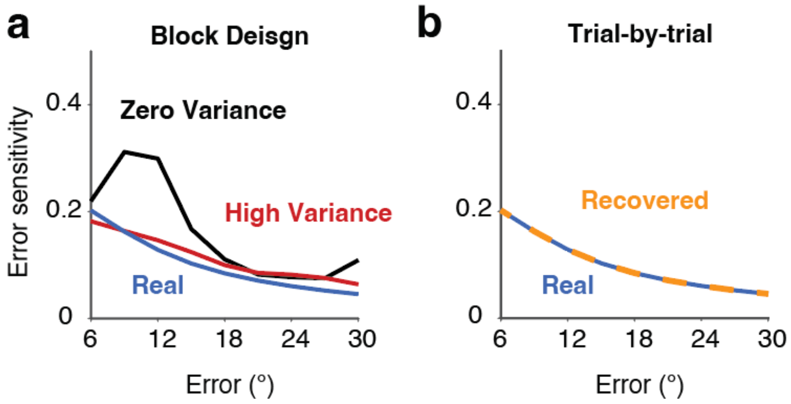

Fig D | The error sensitivity function cannot be recovered in a block design. a) Recovered error sensitivity functions from simulations using a block design in which the perturbation sign is constant. The Real function is based on the curve in Fig 1b, the error sensitivity function used in the simulations. We used this function to simulate data in a block design for high- and zero-variance conditions. We then estimated the error sensitivity function by calculating the trial-by-trial change of hand angle in the simulated data. Using this method, the estimated sensitivity function in the zero variance condition fails to recover the original function. **b)** Similar test using a trial-by-trial design where the direction and the size of the error were randomized across trials (mean perturbation = 0°). Here the recovered error sensitivity function from the simulated data corresponds to the function used in the simulation.

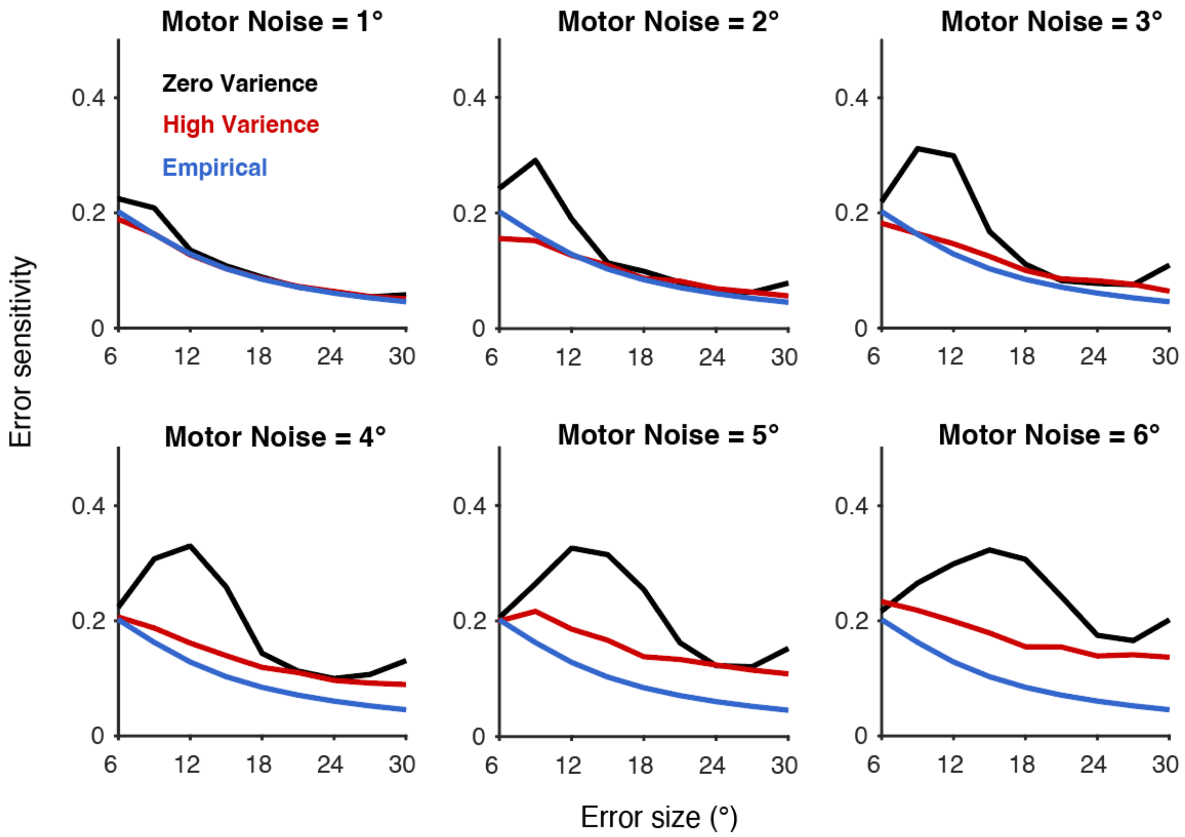

Fig E | The error sensitivity function is not recoverable from data obtained in a blocked design experiment where adaptation will accumulate across trials. The six panels depict the effects for different levels of motor noise (range: 1-6°). Note that the recovered error sensitivity function is always higher in the Zero condition.

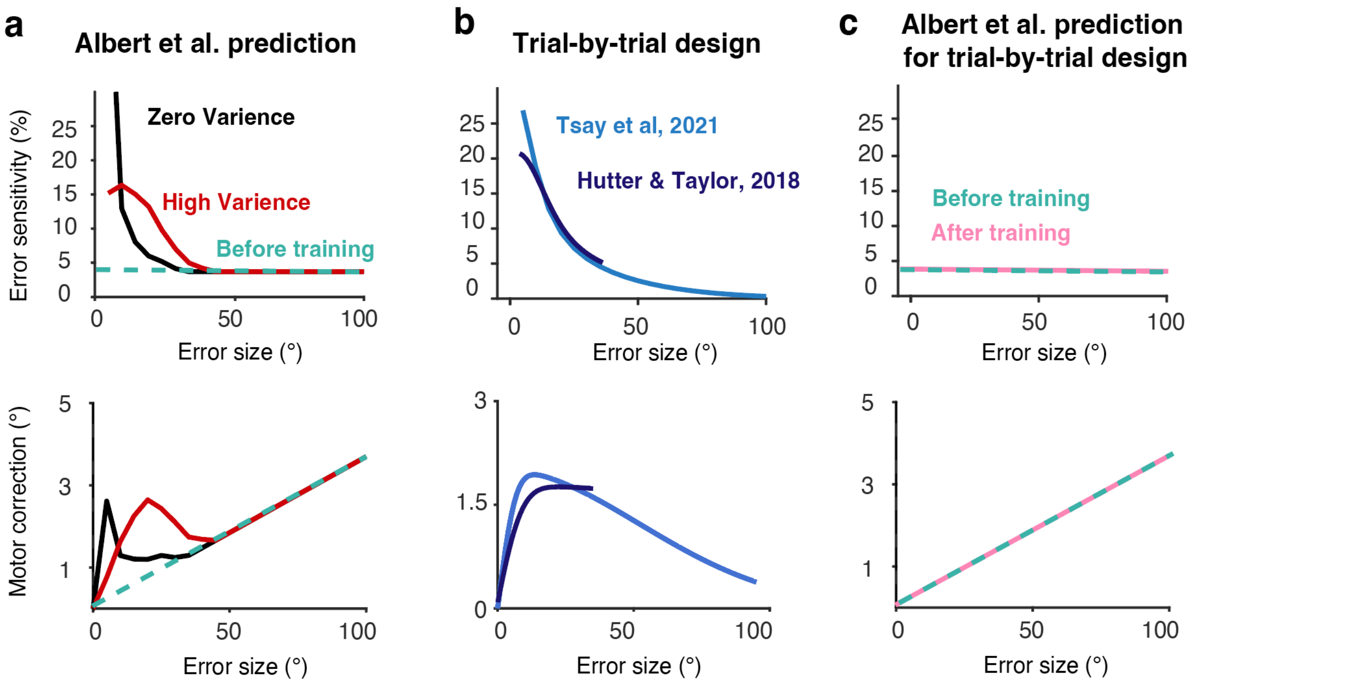

Fig F | The Memory of Errors model fails to predict the error sensitivity function when the perturbation size varies randomly from trial to trial (mean = 0°).

a) Albert et al. assumed that the error sensitivity function is initially a constant (top), resulting in a linear motor correction function (bottom). By itself, this function fails to capture the effect of perturbation variability. In their model, experiencing consistent or inconsistent errors will respectively, increase or decrease error sensitivity. This memory process will result in a non-constant error sensitivity function (top) and a non-linear motor correction function (bottom) for error sizes that were experienced during the experiment (0 to 30°). **b**) Error sensitivity function (top) and the non-linear motor correction function (bottom) estimated from the trial-by-trial designs used by Tsay et al (2021) [7] and Hutter and Taylor (2018) [6]. **c**) In the trial-by-trial design, the MoE predicts that the shape of the error sensitivity function will be maintained over the course of learning given that errors of the same sign and opposite signs will result in no net change in sensitivity. The model fails to match the sensitivity function (top) or motor correction function (bottom).

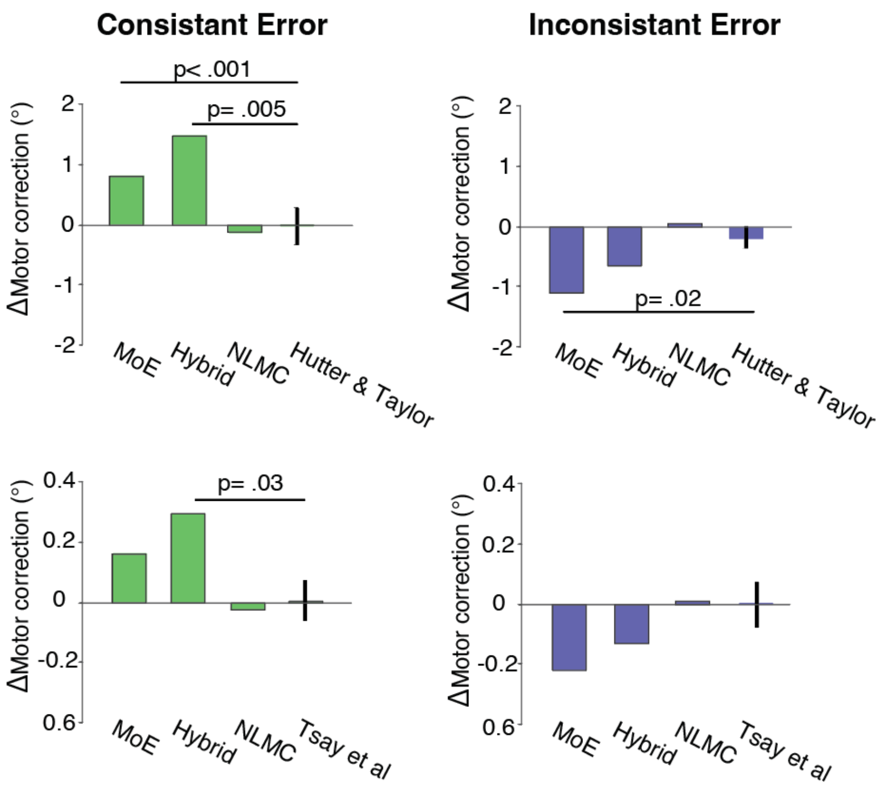

Fig G | Behavioral results obtained in experiments using trial-by-trail design are significantly different from the predicted results based on the MoE and Hybrid models. We compared the magnitude of the motor correction after trial n-1 and after trial n+1 when the two trials had a similar error (differences < 2). The data were grouped based on whether the sign of error on trail n was consistent (left) or inconsistent (right) with the sign of the error on trial n-1. The MoE and Hybrid models predict the size of the motor correction will change after experiencing a similar error on trial n, whereas the NLMC model predicts the size of the motor correction will not change. Re-analyzing the data from two experiments that used a trial-by-trail design [6,8], the size of the motor correction did not change nor differ between consistent and inconsistent trial pair (see also Fig 2). Note that the empirical values are significantly smaller than predicted by the MoE or Hybrid models. The p values are provided for all comparisons showing a significant difference.

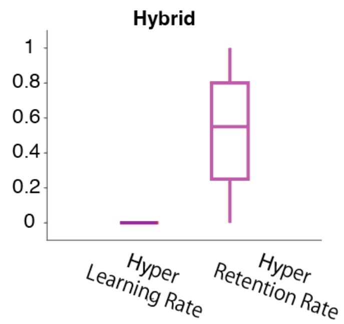

Fig H | Learning rate is not modulated during the learning phase of Experiments 1 and 2. a) We fit the Hybrid model to the bootstrapped (100 times) data set generated from Experiments 1-2. The best fitted hyper learning rate for error sensitivity was zero. As a consequence, the estimated hyper retention rate for error sensitivity is unconstrained and will not influence on the performance of the model.

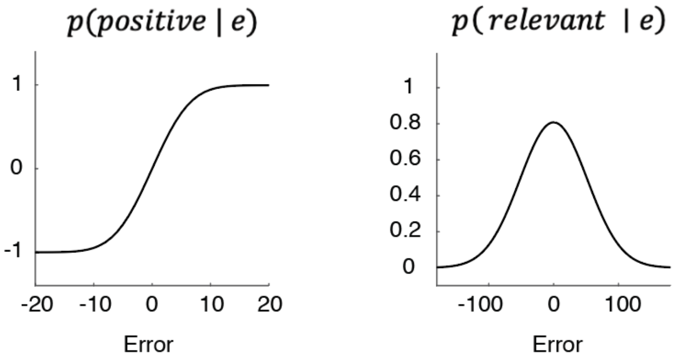

Fig I | Left: Probability of an error being perceived as positive (e.g., clockwise to movement). Right: Probability of error being perceived as relevant to the movement (e.g., resulting from motor noise or miscalibration of the sensorimotor system). The parameters are estimated from Tsay et al. (2021) [7].

Table A: Results of the mixed ANOVA of EXP 1

	**F**	**P**
**Epoch**	F(129,8643)=188.4	<0.001
**Variance**	F(1,67)=8.4	0.005
**Epoch* Variance**	F(129,8643)=1.7	0.07

Table B: Results of the mixed ANOVA of EXP 2

	**F**	**P**
**Epoch**	F(129,9288)=156.1	<0.001
**Variance**	F(1,72)=0.01	0.92
**Epoch* Variance**	F(129,9288)=0.7	1.0
