# Supplementary material for: Perturbation Variability Does Not Influence Implicit Sensorimotor Adaptation
Source: PLoS Comput Biol. 2024 Apr 10;20(4):e1011951. doi: 10.1371/journal.pcbi.1011951 (PMC11034674; doi:10.1371/journal.pcbi.1011951)
Supplement: Attachment — Submitted filename: Reponse2reviewer 17Jan2024.docx [file pcbi.1011951.s002.docx]

**Response to Reviews provided in rejection letter from PLoS Biology (Oct 17, 2023)**

Two reviewers were very satisfied with the revision, although Reviewer 1 did question the novelty of the model. Reviewer 3 raised seemingly substantive concerns with the core assumptions regarding the modeling work and the analyses we performed in this part of the paper, and also had concerns on the novelty front. We address these concerns in the following point-by-point response, first addressing the novelty issue and then the other comments on Reviewer 3. For clarity, we display the reviewer’s comments in blue and our responses in black. We also underscore the three changes we have made in the manuscript in response to reviewer’s comments.

**Novelty issue:**

Reviewer 1: The authors have adequately addressed my comments. Although I still find the scope of the paper limited, notably because the model is really not so new (as "the cornerstone of this model is the extensive literature showing that error sensitivity varies as a function of error size"), this study provides a valuable alternative explanation to the results of Albert et al.

Reviewer 1 states that the “model is really not so new” and highlights that we even cite a large literature describing how error sensitivity varies as a function of error size. But this function, and indeed the model, is not the main point of the paper. Rather, we take this established observation and show how it can explain effects that have taken to reflect sensitivity to context variability in adaptation. The core contribution of this paper is not to test this specific model but to examine the sensitivity of sensorimotor adaptation to error variability. We

present a series of new experiments that provide striking support of the rigidity hypothesis, the novel (and counter-intuitive) constraint we associate with sensorimotor adaptation.

Reviewer 3: The core idea behind this study, that a nonlinearlty in the NLMC response curve would yield mean learning rate estimates that are affected by variance even if learning rate is not in fact directly affected by variance is an astute insightful point that is logically correct. Not to detract from the insightfulness here - but It should be noted that this isn't an entirely original concept, as this core idea here is closely related to the concept of "stochastic resonance", that has interestingly been exploited in a beautiful line of work from a couple decades ago by Jim Collins and colleagues in sensorimotor control where they showed that putting small vibrating devices on the skin to add noise/variability to sensory information could, remarkably, enhance sensory perception by reducing sensory thresholds in individuals with peripheral sensory loss. As a side point, the sensory thresholding nonlinearity present in that case was a thresholding nonlinearity and thus opposite in character (concave up rather than concave down - i.e. having a positive v negative second derivative) compared to the nonlinearity relevant to the current study, which accounts for the opposite effects of variability (increasing sensory perception vs decreasing the amount of learning). The authors may want to consider citing this work and drawing out the interesting parallels to it.

We have not been able to understand how our work relates to stochastic resonance and, as such, don’t follow the relevance of this comment in terms of the novelty of our paper. The non-linear function in stochastic resonance provides a description of the phenomenon that there is an optimal noise level for the detection of an underlying signal. We do manipulate the level of noise, but there is nothing in our model to say that added noise improves sensory thresholds, the core idea of stochastic resonance. Instead, we show that high levels of noise reduce adaptation simply as a statistical consequence of sampling a wider range of error sizes. As such, we do not think our study is related to stochastic resonance, phenomenally or mechanistically.

In sum, neither argument raised about novelty is relevant to the central point of the paper. Most important, we believe the paper will be of considerable interest not just to the motor learning community, but for a broad community of learning theorists since it highlights a very interesting and perhaps unique property of an implicit learning process, one that is essential for ensuring that our movements remain coordinated.

**Point-by-point response to the other comments on Reviewer 3:**

We appreciate the time and effort that Reviewer 3 spent in the review process and the detailed feedback provided. However, we wish to address the reviewer’s comments that suggest we were oblique and unresponsive. We took the reviewer’s comments seriously, and in many cases, used multiple methods to address their concerns. Moreover, it appears that the major concern expressed by the reviewer, namely that we had errors in the opening figure, reflect a misunderstanding on the part of the reviewer.

As mentioned above, i think that the core technical idea behind this study is both correct and astute. So this leaves the issue of whether the nonlinearlty that appears to constitute the NLMC is sufficiently well characterized in the study that it can make meaningful quantitative predictions that explain experimental data. Unfortunately, this is not the case in my opinion.

Overall i'm, unfortunately, rather disappointed by both the revision of the paper and the rebuttal responses to the previous review. Several responses to key concerns from my review and from the the other reviewers seem fairly oblique and thus don't squarely address the heart of the concern. Additionally, there continue to be what appear to be large errors in the Figures, which would mislead readers, just as they misled me for quite some time.

We appreciate that the reviewer finds the core idea of the paper to be “correct and astute.” As noted above, we took the reviewer’s comments very seriously and provided multiple analyses to evaluate how dependent the model was on the assumed NLMC. Specifically, we simulated our model with 1) A normalized function measured in Tsay et al., a method suggested by the reviewer in the original comments; (2) A motor correction function measured in another independent dataset (Hutter & Taylor, 2018); (3) A set of conceptualized motor correction function with different shapes. We do not understand why this would be deemed “unresponsive” and “oblique.”

Error 1: First, panels a and b in figure 1 are highly inconsistent with one another. The caption says that error sensitivity is defined as "the motor correction divided by error size" But the sensitivity values plotted in 1b appear to be about 3 fold, or possibly more, higher that what would be calculated by dividing the motor correction plotted in 1a by the error size. For example, the 30 deg error size corresponds to a motor correction of about 0.33 deg according to figure 1a. That would make the error sensitivity equal to 0.011. but the sensitivity shown in figure 2b for the 30 deg error size is about 0.03 or 0,04, about 3 fold greater than what is should be based on the data in figure 1a. The inconsistency between figures 1a and 1b is not limited to the 30deg point, but rather appears to be present for all values of error.

Error 2: A second inconsistency on the same figure is present when examining 1d and 1e. Figure 1d shows, for the zero variance black line, that the motor correction is between 0.3 and 0.4 deg for errors between 10 and 30 deg, which are the error sizes that would be experienced during early learning for the zero variance condition. But it's fairly clear that the initial slope for the zero variance condition in 1e is greater than 1 deg per epoch, which is about 3 fold greater than the 0.3 to 0.4 deg per epoch values that would be expected based on the plots in 1d or 1a. In sum, it appears that either 1a or 1b is wrong, and also that either 1d or 1e is wrong.

As noted by the Reviewer, there is a 4-fold difference between 1a and 1b. However, this is not an error. 1a is showing empirical data from Tsay et al., 2021; 1b is showing the error sensitivity for these data after scaling the data to match the values reported in Albert et al., 2021. We motivated and described why and how we rescaled the data in the Methods section:

“While the shapes of those functions are similar, the magnitude of the effect is much smaller Tsay et al. This is likely due to the fact that feedback was only presented at the end of the movement and the target was turned off with feedback onset; in most studies, the feedback is continuous and the target remain visible. Given this, we opted to scale the motor correction function such that sensitivity to a 30° error at baseline matched that reported in Albert et al. To accomplish this, we set k=4 for the function derived from Tsay et al. Applying the same logic, we set k=0.4 for simulations using the Hutter and Taylor function, again, seeking to match the baseline sensitivity in response to a 30° error.”

In sum, Fig 1b is constructed by dividing each data point in Fig 1a by the error size and then multiplied by 4. In this way, we use an independent data set to re-evaluate the results from Albert et al to prevent the potential overfitting issue.

Error 2 is essentially the same issue. Here we had to make a choice about which axes to use—scaled or unscaled. We opted to use the unscaled ones (as in Fig 1a) so that the readers could see how the function in Fig 1a would produce the theoretical effects in Figs 1c and 1d.

*We recognize now that Reviewer’s misunderstanding would have been avoided if we had put the scaling factor in the figure caption rather than assume it was sufficient to fully describe the process in the Methods. We have Figure 1a, now using dual axes as a visual guide to describe the scaling factor. We briefly describe this in the revised caption and have elaborated on the justification for the scaling in the Methods section where we describe Model Simulations.*

The key concern expressed in my previous review (strongly related if not precisely stated in both points [1] and [2] and also in the summary paragraph from my previous review) is that the manuscript presented the NLMC model as a single precise prediction, which is highly inconsistent with the fact that the shape of the NLMC curve on which the model is based is not precisely known. As previously pointed out, the data points from Tsay et al 2021 from which the shape of the NLMC curve used in the model was estimated have tremendously wide confidence intervals. These data points +/- SEM are displayed in figure 1a, and even the +/-SEM error bars appear quite wide. The 95% confidence interval error bars, which are a better characterization, would be even wider (about twice as wide for all data points) underscoring that in contrast to the precise NLMC approximation curve displayed in 1c and 1d and the precise NLMC model predictions displayed in panel 1e, confidence in the exact shape of the NLMC curve should be rather low. It's therefore impossible to make a prediction for the NLMC model that would be terribly precise. Despite that fact, the paper presents predictions that are perfectly precise. I believe that this would mislead most readers. I asked in my previous review whether the authors could display 95% confidence intervals for the model predictions, but this request was ignored.. I suspect that, in contrast to the falsely precise model prediction presented in this paper, the range of model predicted that are compatible with the available NLMC data are quite broad - perhaps so broad that the prediction falls largely apart. As it stands the precise predictions presented in this paper are little more than a hunch about what the actual prediction would be if the NLMC were known to a high degree of certainty, and as such does not present compelling evidence for this model. It's possible, of course, that because of internal correlations in the Tsay et al data that the shape of the NLMC is better known than it appears to be based on inspection of 1a (but this is unlikely based on the persistence of wide error bars in the normalized version of the NLMC curve data and fit presented in the rebuttal letter), and with the authors refusal to provide this information a skeptical reader/reviewer would unfortunately have to assume the worst.

[comparatively minor - but related] i take issue with panel d in supplementary figure 2 which is shown in the rebuttal letter in trying to address my comments. The authors claim that this panel shows that a wide variety of NLMC non-linearities can return modelling results that are similar to what they present in the paper. What they fail to say is that the ad hoc collection of non-linear patterns they chose almost all have strong non-linearities that begin even earlier than the data in panel a of that same figure or the data in panels a and b of the preceding figure in the rebuttal letter. This is important because the whole point above about estimating the NLMC well boils down to whether the concave down nonlinearity in it starts early enough (i.e. at sufficiently small errors) and is strong enough. So simulating the results for ad hoc NLMC candidates with earlier rather than later starting points for the nonlineariity isn't terribly helpful. I don't understand why the authors made that choice rather than using much more reasonable NLMC estimates obtained from fits of randomly sampled or bootstrapped subsets of the Tsay et al 2021 data (or other viable data sets).

The reviewer expresses concern that we did not adequately consider the implications of measurement noise associated with the NLMC function used in the model. As mentioned above, we did present a number of approaches on this issue. When coupled with the new experimental data, we believe we had sufficiently addressed the issue. Although the reviewer may disagree with our choices, we think the response and revision were highly transparent.

Given the remaining concern of the reviewer, here we further provided 4 additional lines of evidence/analysis to address the issues raised in Reviewer 3’s comments on the revised manuscript:

2a. As mentioned above, we did not mean to ignore the suggestion to provide 95% confident intervals; we thought we provided a stronger test by using a large range of NLMC functions in simulations, all of which replicated the basic effect of error variance on implicit adaptation. We thought this was stronger since some of the simulated functions fall outside the 95% confidence intervals of the Tsay et al 2021 function, and as such highlight the robustness of the rigidity claim.

We also note that the main purpose of the simulations (with the Tsay function and the set of arbitrary functions) was to show that a non-linear motor correction function can produce the error variance effect observed in Albert et al. As such, the simulations are consistent with a new interpretation of their results, one that challenges the claim that the adaptation process is sensitive to error history. But this is just the starting point of the paper. Our analyses of the published data motivated the new experiments that we conducted, which are presented in the second half of the paper and provide model-free tests of the rigidity hypothesis.

2b. Related to 2a, Reviewer raises a specific concern with our selection of functions in that they all reach the non-linear zone earlier than might be expected and as such, “simulating the results for ad hoc NLMC candidates with earlier rather than later starting points for the nonlinearity isn't terribly helpful.” The reviewer is correct that showing how an NLMC might account for observed differences as a function of error variability does require that the function reach the non-linear zone within the range of errors that will be experienced. If the function is linear across 0-30°, there will not be any difference across low and high variance conditions. However, the range of values we use for the start of the non-linear zone are sufficiently broad and well justified empirically (Tsay et al., 2021, Hutter and Jordan, 2018). When we bootstrap the error correction function from Tsay et al (see below), it always yields a peak between 6-18° (See figure 2c).

With that said, we agree that the set of simulated functions presented in the paper (original and revised manuscript) is somewhat arbitrary. We now consider a more systematic approach, adjusting the shape of the non-linear function and its peak (please note variation in x-axes). As can be seen, while the magnitude of the effect varies, the difference between the high and zero conditions holds regardless of the peak position. The only exception is the condition shown in the lower right, a situation in which the function is essentially linear over the range of errors that would be experienced in the Albert study. We think this analysis will provide readers a clear intuition of how the model works.

*This change is reflected in the revised Figure S2 (now Figure S3).*

2c. Reviewer offers a new recommendation that we use a bootstrapping procedure to establish confidence intervals for the model predictions based on the motor correction function in Tsay et al. We agree that this is a reasonable additional analysis and have now applied this approach. Specifically, we used a bootstrap procedure, taking 24 samples (with replacement) of the data from our pool of 24 participants 200 times. For each bootstrapped non-linear motor correction function, we simulated our NLMC model. In this way, we estimated the 95% confidence interval for the bootstrapped simulations. In the following figure, the left panel shows the 95% confidence interval of the normalized best-fitted motor correction function. To illustrate the range of

peaks, we have normalized the functions based on the peak value. The peak of the function varied from 6-18°. The middle figure shows the 95% confidence intervals for the simulated learning curves for when the experienced error has zero or high variance. As can be seen in the figure, the 95% intervals for the zero and high variance conditions do not overlap. The right panel shows the difference between the two conditions at asymptote. The effect of error variance holds in all of the bootstrapped samples. This new analysis provides a compelling demonstration that the effect of error variability can be attributed to a non-linear motor correction function, a result that holds even given that the estimate of the NLMC is noisy.

*We include the bootstrap analysis as a new supplemental figure (Fig S2).*

We note that when using a bootstrap method to estimate the 95% CI of the asymptotic difference between high and zero in the Albert et al data, the CI was actually greater than in the model simulation (right panel). Given this level of noise in their data, a more precise estimation of NLMC will not enable us to make stronger quantitative inferences. As such, we see the simulations here as demonstrating the plausibility of our alternative interpretation of their results. A more reasonable approach to assess the effect of error variability is to perform new experiments that control the experienced errors. This is what guided our design in Experiments 1 and 2.

2d. Importantly, the paper uses two additional approaches to explore the effect of error variability: 1) Re-analysis of data from an experimental design that looks at trial-by-trial changes in behavior, and 2) two new experiments that directly test the rigidity hypothesis. As stated above, the simulations used to re-evaluate Albert et al. provide a starting point, but the new experiments provide the most compelling evidence that error variability does not change the sensitivity of implicit adaptation. We could reverse the order, starting with the new experiments and then turn to our re-analyses. But we think the current flow is nice and is consistent with the order of how we really conduct the project, first offering a new interpretation of the very interesting experimental results presented by Albert et al, and then presenting new tests to follow up on the rigidity hypothesis.
