# Supplementary material for: Perturbation Variability Does Not Influence Implicit Sensorimotor Adaptation
Source: PLoS Comput Biol. 2024 Apr 10;20(4):e1011951. doi: 10.1371/journal.pcbi.1011951 (PMC11034674; doi:10.1371/journal.pcbi.1011951)
Supplement: Peer review history

# Author comment: Author response to previous submission

**DOI:** 10.1371/journal.pcbi.1011951.r001

**Transfer Alert**

This paper was transferred from another journal. As a result, its full editorial history (including decision letters, peer reviews and author responses) may not be present.

19 Jan 2024

Attachment Submitted filename: Reponse2reviewer 17Jan2024.docx

# Editor report: Decision Letter 0

**DOI:** 10.1371/journal.pcbi.1011951.r002

**Authors:** Barbara Webb, Thomas Serre

27 Feb 2024

Dear Mr Wang,

We are pleased to inform you that your manuscript 'Perturbation Variability Does Not Influence Implicit Sensorimotor Adaptation' has been provisionally accepted for publication in PLOS Computational Biology.

We understand that the manuscript has gone through review at PLOS Biology before transfer to PLOS Computational Biology. The paper has undergone editorial review, and on the basis of the provided response to the previous reviewers, and the revisions made, we consider the current submission clearly meets our criteria for interest and rigor, and are happy to recommend acceptance. 

Before your manuscript can be formally accepted you will need to complete some formatting changes, which you will receive in a follow up email. A member of our team will be in touch with a set of requests.

Please note that your manuscript will not be scheduled for publication until you have made the required changes, so a swift response is appreciated.

IMPORTANT: The editorial review process is now complete. PLOS will only permit corrections to spelling, formatting or significant scientific errors from this point onwards. Requests for major changes, or any which affect the scientific understanding of your work, will cause delays to the publication date of your manuscript.

Should you, your institution's press office or the journal office choose to press release your paper, you will automatically be opted out of early publication. We ask that you notify us now if you or your institution is planning to press release the article. All press must be co-ordinated with PLOS.

Thank you again for supporting Open Access publishing; we are looking forward to publishing your work in PLOS Computational Biology. 

Best regards,

Barbara Webb

Academic Editor

PLOS Computational Biology

Thomas Serre

Section Editor

PLOS Computational Biology

***********************************************************

# Editor report: Acceptance letter

**DOI:** 10.1371/journal.pcbi.1011951.r003

**Authors:** Barbara Webb, Thomas Serre

28 Mar 2024

PCOMPBIOL-D-24-00085 

Perturbation Variability Does Not Influence Implicit Sensorimotor Adaptation

Dear Dr Wang,

I am pleased to inform you that your manuscript has been formally accepted for publication in PLOS Computational Biology. Your manuscript is now with our production department and you will be notified of the publication date in due course.

The corresponding author will soon be receiving a typeset proof for review, to ensure errors have not been introduced during production. Please review the PDF proof of your manuscript carefully, as this is the last chance to correct any errors. Please note that major changes, or those which affect the scientific understanding of the work, will likely cause delays to the publication date of your manuscript. 

Soon after your final files are uploaded, unless you have opted out, the early version of your manuscript will be published online. The date of the early version will be your article's publication date. The final article will be published to the same URL, and all versions of the paper will be accessible to readers.

Thank you again for supporting PLOS Computational Biology and open-access publishing. We are looking forward to publishing your work! 

With kind regards,

Judit Kozma

PLOS Computational Biology | Carlyle House, Carlyle Road, Cambridge CB4 3DN | United Kingdom ploscompbiol@plos.org | Phone +44 (0) 1223-442824 | ploscompbiol.org | @PLOSCompBiol
